# Differential microRNA response to a high-cholesterol, high-fat diet in livers of low and high LDL-C baboons

**DOI:** 10.1186/1471-2164-13-320

**Published:** 2012-07-18

**Authors:** Genesio M Karere, Jeremy P Glenn, John L VandeBerg, Laura A Cox

**Affiliations:** 1 Department of Genetics, Texas Biomedical Research Institute, 7620 NW Loop 410, San Antonio, TX, 78227-5301, USA; 2 Southwest National Primate Research Center, Texas Biomedical Research Institute, San Antonio, TX, 78227, USA; 3 P.O Box 760549, San Antonio, TX, 78245-0549, USA

## Abstract

**Background:**

Dysregulation of microRNA (miRNA) expression has been implicated in molecular genetic events leading to the progression and development of atherosclerosis. We hypothesized that miRNA expression profiles differ between baboons with low and high serum low-density lipoprotein cholesterol (LDL-C) concentrations in response to diet, and that a subset of these miRNAs regulate genes relevant to dyslipidemia and risk of atherosclerosis.

**Results:**

Using Next Generation Illumina sequencing methods, we sequenced hepatic small RNA libraries from baboons differing in their LDL-C response to a high-cholesterol, high-fat (HCHF) challenge diet (low LDL-C, n = 3; high LDL-C, n = 3), resulting in 517 baboon miRNAs: 490 were identical to human miRNAs and 27 were novel. We compared miRNA expression profiles from liver biopsies collected before and after the challenge diet and observed that HCHF diet elicited expression of more miRNAs compared to baseline (chow) diet for both low and high LDL-C baboons. Eighteen miRNAs exhibited differential expression in response to HCHF diet in high LDL-C baboons compared to 10 miRNAs in low LDL-C baboons. We used TargetScan/Base tools to predict putative miRNA targets; miRNAs expressed in high LDL-C baboons had significantly more gene targets than miRNAs expressed in low LDL-C responders. Further, we identified miRNA isomers and other non-coding RNAs that were differentially expressed in response to the challenge diet in both high LDL-C and low LDL-C baboons.

**Conclusions:**

We sequenced and annotated baboon liver miRNAs from low LDL-C and high LDL-C responders using high coverage Next Gen sequencing methods, determined expression changes in response to a HCHF diet challenge, and predicted target genes regulated by the differentially expressed miRNAs. The identified miRNAs will enrich the database for non-coding small RNAs including the extent of variation in these sequences. Further, we identified other small non-coding RNAs differentially expressed in response to diet. Our discovery of differentially expressed baboon miRNAs in response to a HCHF diet challenge that differ by LDL-C phenotype is a fundamental step in understating the role of non-coding RNAs in dyslipidemia.

## Background

Cardiovascular disease (CVD), a leading cause of mortality in the United States, is commonly due to the development of atherosclerosis [1]. Atherosclerosis is a chronic inflammatory disease affecting medium and large arteries, resulting from a complex interaction between genotype and environmental factors including diet, which influences dyslipidemia. Clinical and epidemiological studies indicate that atherosclerosis is positively correlated with serum LDL-C and inversely correlated with high-density lipoprotein cholesterol (HDL-C) [2-5], demonstrating that increased levels of serum LDL-C is a major risk factor for atherosclerosis [6]. However, the exact mechanism by which LDL-C promotes atherosclerosis remains an active aspect for research. Early studies by Brown and Goldstein [6] demonstrated that oxidized LDL-C (ox-LDL-C) is a prerequisite for initiation of atherosclerotic lesions, leading to the development of intermediate and complex plaques [7,8]. Subsequent studies have confirmed that ox-LDL-C is an essential atherosclerotic risk factor, triggering arterial cellular modification [9-11]. Lowering LDL-C reduces the risk for atherosclerosis in humans, and modification of diet and lifestyle, and administration of drugs is a major strategy to control CVD [12].

Apart from the physiological characteristics, understanding the genetic mechanisms underlying dyslipidemia and subsequent development of atherosclerosis is fundamental to therapeutic interventions. Many genetic factors influencing risk factors for atherosclerosis have been identified [13], including genes underlying the vulnerability of arterial intimal layer to ox-LDL-C [10,14]. The expression of genes important for development of atherosclerosis is modulated by a variety of elements, including microRNAs (miRNAs).

miRNAs are small endogenous non-coding RNAs that are evolutionarily conserved across many species and the biogenesis of miRNAs has been described [15,16]. miRNAs down-regulate gene expression by translational repression [15], degradation and deadenylation [17,18]. Occasionally miRNAs can also up-regulate gene expression [19,20]. These small non-coding RNAs have been implicated in a range of biological processes, including cell growth, differentiation, apoptosis, and cholesterol metabolism [15,21]. These processes are important for the initiation and progression of atherosclerosis. Consequently, dysregulation of miRNA function may lead to atherosclerosis. For example, miR-365 is up-regulated by ox-LDL-C, leading to reduced expression of *BCL2*, an antiapoptotic gene, in arterial endothelial cells [22]. miR-155 and miR-146a are associated with accumulation of ox-LDL-C in monocytes [23,24]. It has been shown that miR-92a controls angiogenesis of atherosclerotic plaques in mice, leading to destabilization and rupture [25]. miRNAs, let-7 and miR-17-92 cluster, are known to target thrombospondin-1, an inhibitor of angiogenesis [26]. Furthermore, miR-210, -15b, -16, and -20b are implicated in down-regulation of vascular endothelial growth factor [27-29], a key gene in cell migration in the atherosclerotic lesion. Studies also indicate that the up-regulation of miR-335 and −122 is associated with lipid metabolism in obese mice [30,31] and chimpanzee [32]. This suggests that expressed miRNAs play a fundamental role in dyslipidemia, a major risk factor for atherosclerosis. Importantly miRNAs have been shown to exhibit temporal and spatial expression patterns, emphasizing the need to define cell–type–specific miRNA expression profiles, which require expression-sensitive tools to capture low-abundance small RNAs.

Although the baboon is a well-characterized model for human biomedical studies, including CVD, very little is known about baboon (*Papio hamadryas*) miRNAs [16]. Discovering liver miRNAs, identifying diet responsive miRNAs that differ between low and high LDL-C responders, and understanding how miRNAs regulate gene expression in the baboon will provide insights on the role of miRNAs in dyslipidemia. In this study, we have used Next-Generation sequencing methods to perform deep sequencing of small RNA libraries derived from liver biopsies collected before and after a HCHF challenge diet from six half-sib baboons (low LDL-C, n = 3; high LDL-C, n = 3), differing in their LDL-C response to dietary fat and cholesterol. The sequence reads were aligned to human genome; notably the current draft of baboon genome is an unannotated draft assembly which does not provide chromosomal coordinates or transcript identification as with the human genome sequence. However, based on physical maps, the baboon and human genomes exhibit considerable sequence conservation and synteny [33]. We identified differentially expressed miRNAs in response to the challenge diet in the two LDL-C phenotypes and used *in-silico* methods to identify miRNA targets.

## Results

### Annotation of small RNAs and identification of novel miRNA genes

Sequencing 12 baboon liver small RNA libraries yielded a total of 2,765,191 sequence reads with an average 230,433 reads per sample ranging from 95,728 to 441,911 reads. A total of 770,816 unique tags mapped perfectly to the human genome sequence, with an average of 64,235 tags per sample ranging from 38,701 to 138,915 tags. On average 7% of the tags per sample were identical to human miRNAs. Table 1 shows the number of clusters generated, number of mapped unique tags per sample, and the proportion of tags mapped to miRNA sequences and their expression levels.

**Table 1:** Baboon liver small RNA libraries: number of clusters, sequence tags and their expression levels

**Phenotypes**	**Diet/Half-sib IDs**	**No. of Clusters**	**Mapped unique tags**	**% tags mapped to human miRNAs**	**% miRNA expression**
Low LDL-C					
	Chow				
	6910	192,812	138,915	3	61
	6265	338,750	43,778	4	48
	8024	127,616	79,038	7	67
	HCHF				
	6265	441,911	38,701	5	35
	8024	210,084	41,087	6	63
	6910	368,270	74,531	7	63
High LDL-C					
	Chow				
	8623	281,930	47,782	5	65
	1X3817	171,740	50,363	8	68
	1X4154	162,244	38,943	7	67
	HCHF				
	8623	217,753	41,587	5	63
	1X3817	95,728	78,928	7	65
	1X4154	156,353	97,163	15	74
	Average	230,433	64,235	7	62
	Total	2,765,191	770,816		

**Figure 1:**
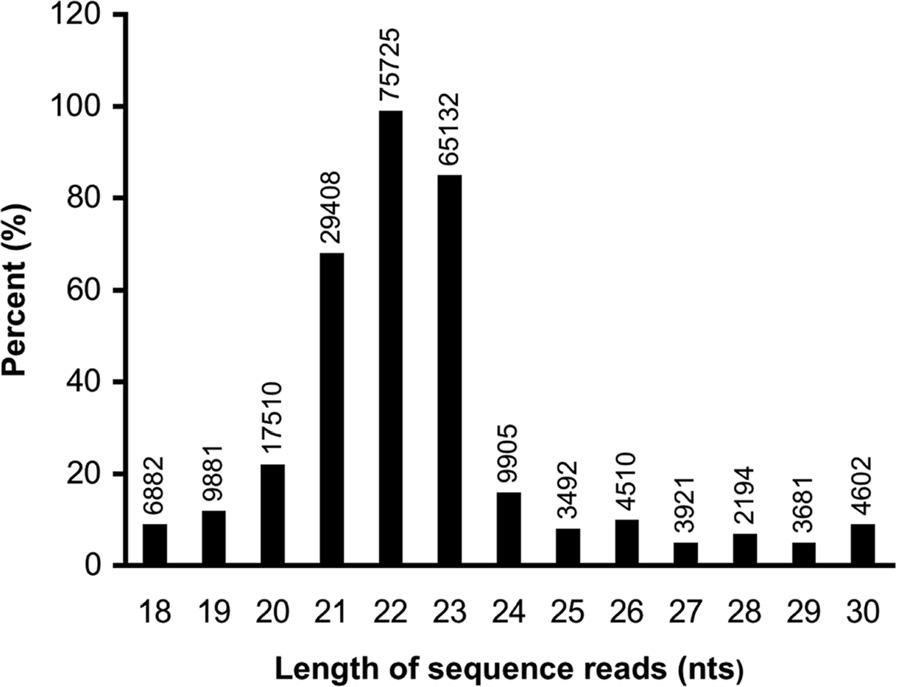
**Frequencies and lengths of baboon sequence reads.** The proportion (%) of different lengths of sequence reads that aligned to the human miRNAs. The number above each bar indicates the total number of reads with corresponding size.

**Figure 2:**
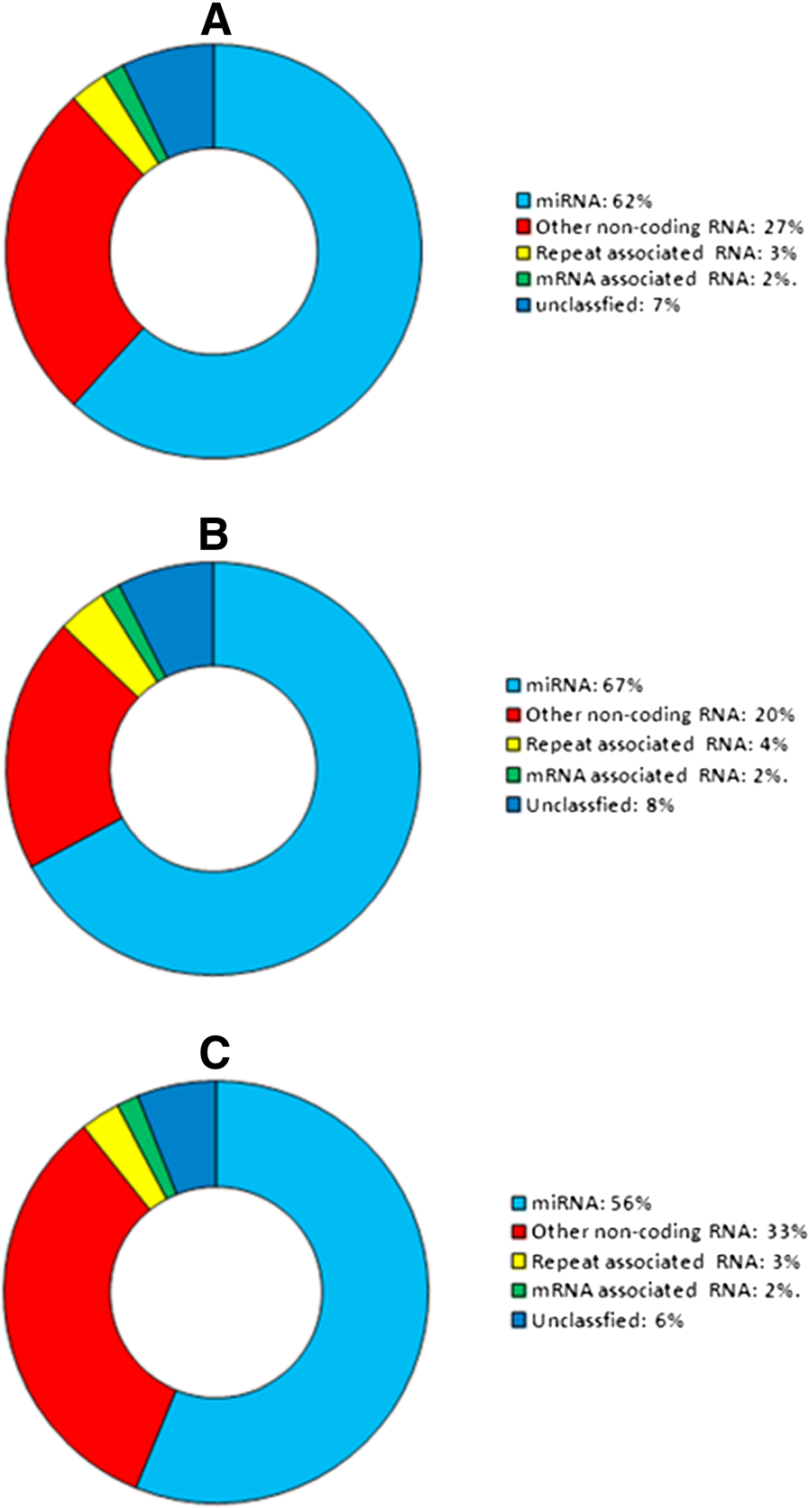
**Frequency of small RNAs in a) combined low and high LDL-C, b) High LDL-C only and c) Low LDL-C only liver samples.** The proportion of expressed baboon small RNAs identical to human: i) miRNAs are shaded light blue, ii), other non-coding RNAs (including snoRNAs, tRNAs, siRNAs, and snRNAs) are shaded red, iii) repeat-associated RNA sequences are shaded yellow, and iv) coding transcript-associated sequences are shaded green. The proportion of mapped but unclassified sequences is shaded dark blue.

The small RNA libraries exhibited a diverse size distribution of sequence reads that aligned to human genome (Figure 1). Sequence reads (22nts) were the most abundant in all sequenced libraries. The proportion of expression levels of small non-coding RNAs is shown in Figure 2. miRNAs were the most abundantly expressed small RNAs (mean = 62%) in libraries from low and high LDL-C baboon livers (Figure 2a). Other small non-coding RNAs such as small interfering RNAs (siRNAs), small nucleolar RNAs (sno RNAs), small nuclear RNAs (snRNAs), and transfer RNAs (tRNAs) comprised 27% of the total expressed tags. Repeat-associated RNAs accounted for only 3% of the tags. Unique tags that did not map (“unclassified”) to any small non-coding RNA databases or to the human genome comprised approximately 7% of the small non-coding RNAs expressed in the baboon livers. Figure 2b and 2C show the distribution of expression levels of small RNAs detected in low LDL-C and high LDL-C baboons. The mean proportion of miRNAs expressed in the high LDL-C baboon livers (67%) was higher than in low LDL-C (56%) (Figure. 2b and 2C).

We discovered 517 miRNAs in baboon livers: 490 were identical to human miRNAs, herein referred to as known miRNAs, and 27 novel baboon miRNAs (Table 2). Each novel miRNA had a corresponding near complementary sequence, referred as miRNA star (miRNA*). Table 3 shows the total numbers of known and novel miRNAs detected from low and high LDL-C baboons on chow and HCHF diets. An average of 398 known miRNAs was detected in the 12 samples ranging from 379 to 442 miRNAs per sample. A higher number of novel miRNAs was identified in high LDL-C responders compared to low LDL-C responders (low LDL-C, n = 20; high LDL-C, n = 29). Further, we identified more novel miRNAs in baboon livers from the HCHF challenge diet than chow diet in high LDL-C responders. Table 4 shows the breakdown of miRNAs expressed in each baboon phenotype in response to the two diets. In both baboon phenotypes, the majority of known miRNAs (89%) were not diet specific, while 11% of the miRNAs were expressed only in response to a specific diet. The HCHF diet elicited expression of more miRNAs in high LDL-C responders than in low LDL-C responders (Fisher’s exact test; p = 0.0001). In contrast low LDL-C responders expressed more miRNAs on chow diet (p = 0.0001) than the HCHF diet. Thus, miRNA expression is responsive to diet and potentially associated with dyslipidemia in baboons. Additional files 1 and 2 contain known and novel miRNAs detected from all the liver samples.

**Table 2:** miRNAs expressed in baboon livers

**Phenotype**	**Identical baboon-human miRNAs**	**Novel baboon miRNAs**
Low LDL-C	33	5
High LDL-C	19	12
Common to low and high LDL-C	438	10
Total	490	27

**Table 3:** Frequency of miRNA expression for each LDL-C phenotype and each diet (n = 3 per group)

**Phenotype/Half-sib IDs**	**Identical baboon-human miRNAs (mean 397.50 ± 19.82)**		**Novel baboon miRNAs (mean 4.08 ± 1.98)**	
	**Chow**	**HCHF**	**Chow**	**HCHF**
High LDL-C				
8623	409	425	7	8
4145	381	407	3	3
3817	379	387	4	4
Total	1169	1219	14	15
Average	390	406	5	5
Low LDL-C				
6265	391	396	4	3
8024	383	379	1	2
6910	442	391	5	5
Total	1216	1166	10	10
Average	405	389	3	3

**Table 4:** miRNA expression categorized by diet for each LDL-C phenotype

	**Identical baboon-human miRNAs**	**Novel miRNAs**
Low LDL-C		
Chow only	37	4
HCHF only	14	3
Both diets	420	3
Total	471	10
High LDL-C		
Chow only	16	5
HCHF only	34	7
Both diets	417	5
Total	467	17

### Differential expression profiling of miRNAs responsive to dietary fat

There was a statistical difference (Fisher’s exact test; p < 0.05) between the number of miRNAs differentially expressed in high LDL-C baboons in response to HCHF diet (n = 18) compared to 10 miRNAs for low LDL-C responders. Two miRNA families are shared between the two baboon phenotypes. In this study miR-29 family members were significantly down-regulated in response to HCHF diet in both low and high LDL-C baboons. Interestingly, the expression of some miRNA gene families showed polycistronic regulation, such that a subset of members of one family was either down-regulated in one phenotype or up-regulated in another. For example, a subset of miRNA let-7 family members was down-regulated in low LDL-C baboons but up-regulated in high LDL-C baboons (Additional file 1). In the Additional file 3: Table S1 lists the miRNAs that were differentially expressed, p-value, fold change and the complete mature miRNA sequences.

Moreover, some miRNAs were strictly diet-specific; these miRNAs, although not differentially expressed, exhibited peculiar characteristics in which they were expressed either in response to chow diet but not HCHF diet, or *vice versa* (Additional file 3: Table S2). In addition some miRNAs were polymorphic between low and high LDL-C baboons. For example, miR-181c and 302a were expressed in low LDL-C baboon livers while 181a and 320b were detected in high LDL-C baboon livers.

We observed that a substantial portion of sequence tags perfectly mapped to other non-coding RNAs including snoRNA, siRNA, snRNAs, and tRNAs in Rfam database. These RNAs accounted for 20-33% of the total expressed tags in the two phenotypic groups (Figures 2b and 2C). Moreover, 31% (1,064/3,456) of other non-coding RNAs in high LDL-C baboons were differentially expressed between chow and HCHF diets (False Discovery Rate (FDR) ≤ 0.05) (Additional file 4). These small RNAs were not differentially expressed in low LDL-C baboon livers in response to the HCHF diet.

### Targets of differentially expressed miRNAs in baboon livers

miRNA target predictions indicate many-genes- to -many-miRNAs regulation modules in which one miRNA has multiple targets, and a protein coding gene has multiple miRNA sites. A significantly higher number of targets were predicted for miRNAs differentially expressed in high LDL-C livers compared to low LDL-C livers (Fisher’s exact test; p = 0.001) (Table 5). miRNAs common in both baboon phenotypes accounted for 13% (2,931/22,598) of the total human gene targets and about 80% (111/140) of the experimentally validated targets. We found that each differentially expressed miRNA family had on average 2,124 target genes ranging from 40 to 3,586. A substantial number of rhesus targets overlapped with those in human genome. Because the currently available baboon genome is an unannotated draft sequence we were not able to perform comparative analyses with baboon and rhesus and baboon and human genomes. Furthermore, the draft baboon genome sequence is not available via the TargetScan software, Identified miRNA targets included genes that have been implicated in risk factors contributing to atherosclerosis. These targets include protein coding genes involved in lipid metabolism, fatty acid biosynthesis, and immune and inflammatory systems. We estimate that 9% (1,357/14,356) of the miRNA targets comprise CVD-related genes in human and/or in mouse.

**Table 5:** Number of predicted and validated gene targets for the 28 differentially expressed miRNA families

	**Low LDL-C**	**High LDL-C**	**Common**
miRNAs families differentially expressed in response to HCHF diet, p ≤ 0.05	10	18	2
Human gene targets	9,764	12,834	2,931
Rhesus gene targets	9,367	12,164	2,651
Overlap between rhesus and human genes	8,447	11,516	2,403
Experimentally validated targets	16	124	111

Novel miRNAs expressed in high LDL-C livers, regardless of diet, had a greater number of predicted target genes (n = 2,448) than miRNAs expressed in low LDL-C livers (n = 2,047) (Table 6). Of the 2,448 genes targeted by novel miRNAs from high LDL-C livers, 99% (2,438/2,448) are predicted to be targeted by known miRNAs expressed in high LDL-C baboon livers. Interestingly, 80% (1,631/2047) of miRNA targets in low LDL-C baboons are predicted to be regulated by miRNAs responsive to chow diet. These results suggest that liver-expressed miRNAs in high LDL-C baboons potentially target genes responsive to elevated LDL-C concentrations while in low LDL-C responders, the majority of miRNA targets are responsive to lower concentrations of LDL-C.

**Table 6:** Novel baboon miRNAs categorized by diet and LDL-C. The numbers of predicted target genes, and the 2-7mer seed region for each novel miRNA, are shown

Chow diet			
High LDL-C		Low LDL-C	
‘Seed’	No. of targets	‘Seed’	No. of targets
GGGTGCG	37	ACCTCGA	6
TAAGAAC	423	TGGAATA	315
TGGACGT	8	TTATAAT	826
TTGACTA	111		
Total unique targets	561	Total unique targets	1079
HCHF diet			
High LDL-C		Low LDL-C	
‘Seed’	No. of targets	‘Seed’	No. of targets
CCCAAAT	306	TTCTGAT	441
CCCGCGG	6	TGGACGT	8
AGAGCTT	575	GCGCTGG	15
TTTTGCT	149		
ACCTCGA	6		
CTACCCC	59		
CTGTGAG	14		
Total unique targets	902	Total unique targets	416
Both diets			
High LDL-C		Low LDL-C	
‘Seed’	No. of targets	‘Seed’	No. of targets
ATCAAGG	147	ATCAAGG	147
CCGCCCT	14	CCGCCCT	14
TTTTTGC	349	CAGGCAC	402
CAGGCAC	402		
TTGTTGC	179		
Total unique targets	985	Total unique targets	552

## Discussion

### Baboon and dyslipidemia

High LDL-C is the major risk factor for atherosclerosis, the leading cause of CVD. Underlying the pathogenesis of atherosclerosis are complex networks of genes regulated by a plethora of elements including miRNAs. In this study we investigated differential expression of hepatic miRNAs in baboons differing in serum LDL-C concentrations in response to HCHF diet, and predicted miRNA targets. Liver is a major organ involved in lipid metabolism and regulation of serum cholesterol including LDL-C, and hepatic inflammation is associated with early atherosclerosis [34]. To understand the liver microRNAome relevant to LDL-C phenotype and dietary fat and cholesterol, we performed high coverage Next-Generation sequencing, annotated the baboon liver small RNA transcriptome and quantified expression of small RNAs. The baboon is a well-characterized model for dyslipidemia and atherosclerosis [35,36], and is very similar to humans both physiologically and genetically [33]. In addition to genetic background, we are able to control diet and environment in a nonhuman primate, which is not feasible in humans. For this study, animals exhibiting discordant LDL-C serum measures (low and high LDL-C responders) were challenged for 7 weeks with a HCHF diet. Liver samples were collected from six half-sibs discordant for LDL-C before and after the challenge diet. By analyzing half-sib baboons discordant for the quantitative trait (< 2 S.D. difference), we minimized genetic variation due to genetic background and increased the likelihood that we would discover miRNAs that are diet responsive and that differ by LDL-C phenotype.

### Identification of baboon liver miRNAs

The distribution of non-coding RNA sequences identified in this study is in concordance with previous studies identifying miRNAs using deep sequencing methods. The length distribution of sequence reads indicates that 22-nt fragments were the most abundant (Figure 2), corresponding to the average size of mature miRNAs [37]. We identified various categories of small non-coding RNAs from baboon liver samples and observed that miRNAs were the most abundant (62%), (Figure 2a), in concordance with previous studies [38,39].

Various methods were applied in the identification of baboon miRNAs. The first study to identify baboon miRNAs employed a combined approach involving genomic computation and microarray expression [16]. In our present study, we identified 490 miRNAs identical to human miRNAs. This number far exceeds the 227 liver miRNAs identified by genomic computational prediction. Moreover, the total number of expressed baboon liver miRNAs identified by sequencing increased by 21% that detected by microarray [16]. In addition we identified 27 novel baboon miRNAs with corresponding complimentary (‘star’) sequences (Table 2). The presence of star miRNAs is compelling evidence for the DICER-like processing from a miRNA hairpin and stringency measures applied in the analysis. The identified baboon miRNAs will contribute to the species-specific miRNA database. These results demonstrate the power of parallel and high-throughput Next-Generation sequencing in elucidating differential expression profiles of miRNAs in baboon livers.

Next Generation (NG) deep sequencing has become a method of choice for discovering miRNA sequences and expression. NG technology allows detection of low copy number miRNAs in the transcriptome and discovery of novel small RNAs, with excellent reproducibility [40]. Although QRT-PCR is the method of choice for validating array-based RNA expressions, it is a relative measure of expression normalized against expression of a ‘house-keeping’ gene. In contrast, expression profiling by deep sequencing is normalized against the whole transcriptome providing more robust data in a high coverage sequencing experiment than what can be attained by QRT-PCR. For example, in this study the average read counts for baboon liver small RNAs was approximately 800,000. Moreover, NG sequencing enables detection of variation in mature miRNAs and RNA editing. Thus, deep sequencing remains an extremely sensitive approach for identifying miRNA expression levels and for detecting novel and variant miRNA sequences.

### Expression of small non-coding RNAs

**Figure 3:**
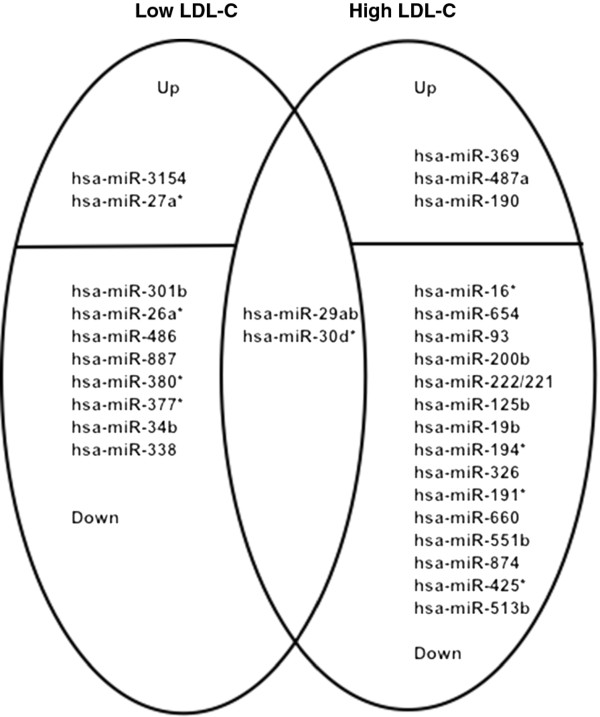
**Families of baboon liver differentially expressed miRNAs.** The Venn diagram includes both up- and down-regulated miRNAs (p ≤ 0.05) in low and high LDL-C baboon responders in response to the HCHF diet challenge.

Expression profiling of non-coding RNAs in baboon livers indicated that a subset of miRNAs is diet-specific, potentially influencing LDL-C variation in baboons. A significantly higher number of miRNAs were expressed in response to HCHF challenge diet in high LDL-C baboons than in low LDL-C baboons (Table 3). In addition we have discovered miRNAs differentially expressed in response to the HCHF diet and common in both low and high LDL-C baboons (Figure 3).

Several expressed miRNAs in this study were previously reported to be involved in lipid metabolism and CVD, including atherosclerosis (Table 7). For example, miR-122, which is highly abundant in liver, is associated with serum cholesterol levels in mice and primates [30,32]. miR-221 and miR-222, which share an identical seed region, are associated with arterial smooth muscle angiogenesis [41]. Moreover, down-regulation of miR-155 and -146a is associated with enhanced accumulation of oxidized LDL-C in monocytes [23], potentiating inflammation of the intima layer of arteries and generation of atherosclerotic lesions. Further, increased expression of miR-335 is associated with elevated lipid metabolism in obese mice [31]. Our results show that miR-122 is up-regulated in response to the HCHF challenge diet in low LDL-C but down-regulated in high LDL-C baboons. miR-221 and miR-222 were significantly down-regulated in response to HCHF diet in both low and high LDL-C baboons. miR-155 and -146a were down-regulated in response to the HCHF diet in our study. Further we observed that miR-335 is up-regulated in low LDL-C baboons but is not expressed in high LDL-C animals. This may indicate that expression of some miRNAs may be dependent on the threshold level of serum cholesterol. This observation is consistent with results by Maxwell et al. [42], which indicate that reduced serum cholesterol levels may trigger expression of some genes involved in lipid metabolism. Altogether, the results from the sequence data indicate we were able to capture some of the potential miRNA candidates encoding risk factors related to atherosclerosis.

**Table 7:** Expression profiles of miRNAs associated with metabolic disorder risk factors

**miRNA**	**Low LDL-C (Chow vs HCHF)**	**p value**	**High LDL-C (Chow vs HCHF)**	**p value**	**Biological processes associated with disease risk factors**	**References**
miR-29b	Down	0.018	Down	0.01	Demethylation of MMP-2/MMP-9 genes involved in cell migration in aorta	[43]
miR-26a-1*	Down	0.007	Down	0.32	Regulator of vascular smooth muscle cell function	[44]
miR-221	Down	0.45	Down	0.02	Angiogenesis in hypertrophy	[41]
miR-222	Down	0.39	Down	0.01	Angiogenesis in hypertrophy	[41]
miR-222*	Up	0.98	Down	0.66		
miR-143	Down	0.67	Down	0.17	Proliferation of vascular smooth muscle cells	[45]
miR-122	Up	0.69	Down	0.19	Lipid metabolism	[30,32]
miR-122*	Down	0.43	Down	0.38		
miR-335	Up	0.48	-	-	Lipid metabolism	[31]
miR-335*	Down	0.31	Down	0.29		
miR-21	Down	0.63	Down	0.62	Cell proliferation in carotid artery	[46]
miR-1	Down	0.42	Down	0.35	Repolarization and diastolic activity	[47]
miR-146a,b	Down	0.12	Down	0.36	LDL-C oxidization accumulation in monocytes	[23][24]
miR-155	Down	0.17	Down	0.43	Oxidized LDL-C accumulation in monocytes	[24]
miR-130b	Down	0.98	Down	0.59	Arteriosclerosis obliterans	[48]
miR10a	Down	0.39	Down	0.47	Regulation of pro-inflammation in aorta	[49]

- Indicates that the miRNA was not detected. miRNA stars (*) are presented for comparison with mature miRNAs.

Although miR-33a/b were not differentially expressed in response to the HCHF challenge diet both miRNAs were detected in low and high LDL-C livers, however, at differing amplitudes. miR-33a was more abundant in low LDL-C while miR-33b was more abundant in high LDL-C livers. These findings are consistent with previous studies that indicate miR-33a is highly expressed in low sterol condition whereas miR-33b is more abundant in cholesterol-filled cells [50]. miR-33 family is transcribed from the introns of sterol-regulatory element-binding factor (*SREBF*) isoforms and is linked to cholesterol homeostasis [51].

In this study, we identified differentially expressed miRNAs that are predominantly engulfed by HDL particles [52]. miR-877 and 191 were differentially expressed in low and high LDL-C livers, respectively. The results are consistent with findings by Vickers and colleagues, indicating that miR-877 is abundantly expressed in normal individuals while miR-191 is up-regulated in familial hypercholesterolemia (FH) human subjects. Mutation in LDL receptor (*LDLR*) leads to FH and severe development of atherosclerotic lesions in homozygous individuals [53]. In addition we found that miR-19 was differentially expressed in high LDL-C baboons and not in low LDL-C responders. miR-19 is abundant in *LDLR* mutant mice [52]. Together, these findings indicate that these miRNAs are potentially involved in regulation of dyslipidemia and HDL particles may be involved in their transport to recipient cells as an anti-atherogenic mechanism.

During the biogenesis of miRNAs, a duplex RNA is generated [15]. Only a single strand (mature miRNA) is loaded into RNA-induced silencing complex (RISC) while a near complementary strand, miRNA star (miRNA*), is degraded [54,55]. Recent studies based on deep sequencing have uncovered the presence of miRNA* in different systems but in low abundance [38]. This indicates the power of deep sequencing in detecting low copy number miRNAs previously thought to be degraded. In our study we confirmed presence of miRNAs* (Additional file 1). In addition we discovered that some miRNAs* exhibit higher expression than the mature miRNAs, are inversely regulated between low and high LDL-C responders, or are detected in some samples where mature miRNAs are absent. For example, miR-222* was up-regulated in response to HCHF diet in low LDL-C baboon livers but down-regulated in high LDL-C responders while mature sequences were down-regulated in both LDL-C phenotypes. These observations indicate that processing of miRNAs is regulated and that regulation of processing can affect gene expressions.

Although miRNA families have a consensus 5’ end ‘seed’ region, little is reported on upstream polymorphisms, which may affect the binding efficiency of the miRNA on mRNA UTRs, in relation to the phenotype of interest. In this study we identified some miRNAs that have nucleotide polymorphisms between low and high LDL-C responders including miR-181 and 302. miR-181 variants have identical ‘seed’ region; however, variant 181a has a G allele insertion flanking the 8-mer seed region in comparison to 181c. miR-181 has a consensus binding site in humans, chimpanzee and rhesus macaque but not mice and rats. In comparison, 302a and c have G/A substitutions at the 3’ end, contributing to differing total context scores and binding efficiency. Mice, rats, rhesus macaque, chimpanzee and humans have a consensus binding sequence for miR-302. In contrast rabbits, which are often used to model dyslipidemia and atherosclerosis do not have sites for miR-181 or 302 [56].

Furthermore, differentially expressed miRNAs in baboon livers (Figure 3) have been implicated to have multiple roles in different types of diseases. For example miR-16 is reportedly over-expressed in lung cancer [57], down-regulated in prostate cancer [58] and up-regulated in rheumatoid arthritis [59]. Down-regulation of miR-34b is associated with tumorogenesis suppression [60] and Parkinson’s disease [61] and its suppression via methylation confers anti-apoptotic effects on colorectal cancer [62]. miR-34b targets include *TENC1* (tensin like C1 domain containing phosphatase), which has been implicated in apoptotic events in vitro [63]. These observations suggest that miRNAs have different roles in distinct tissues and that the effects on gene expression should be interpreted in the context of cell state.

Our results indicate that a significant proportion of expressed small non-coding RNAs include other miRNA-sized small non-coding RNAs such as snoRNAs, siRNAs, snRNAs and tRNAs. Non-coding RNAs (n = 3,455) were expressed in high LDL-C responders while 4,645 were identified in low LDL-C livers. We observed that 31% (1,064/3,455) of other non-coding RNAs in high LDL-C baboons were differentially expressed (FDR ≤ 0.05) in response to HCHF diet. Non-coding RNAs were not differentially expressed in low LDL-C baboons. A total of 2,277 RNAs were expressed in both baboon phenotypes including 99% of those differentially expressed in high LDL-C livers. The functional role of these small RNAs in dyslipidemia is not clear. However, prior reports indicate that deep sequencing generates a considerable number of sequence tags perfectly mapping to other known small non-coding RNAs [64]. At least one study [65] identified snoRNAs with miRNA-like function and DICER processing signatures. It is plausible these RNAs may have functional roles in dyslipidemia.

miRNAs are often expressed in gene clusters, and their transcription is thought to be closely linked [16,66]. In this study we observed that miRNA clusters and cluster members were coordinately regulated. For example, the majority of let-7 family members are down-regulated in low LDL-C responders but up-regulated in high LDL-C responders. However, not all members of the family exhibited a coordinated pattern, suggesting an independent regulation event. A recent study [38] confirmed that some miRNAs show differential expression in distinct cells due to post-transcriptional regulation associated with variation in the levels of DICER, an enzyme involved in biosynthesis of mature miRNA. It is plausible that the expressions of some let-7 cluster members are dependent on cell context or independent of cluster transcription unit. Recent studies have uncovered the association of clustered miRNAs and complex diseases including B-cell leukemia [67,68] and testicular carcinoma [69-71]. These miRNA clusters tend to occur in fragile regions of genomes and may be species-specific [16,72,73].

### miRNA gene targets and atherosclerosis

Many of the predicted targets of expressed miRNA family members, with identical seed regions, overlapped considerably. Thus, it is logical to focus on identical seed regions or the miRNA family names when considering targets of miRNAs; miRNAs with same seed region are classified as one family. In this study, we sought to identify genes targeted by expressed miRNA families in low and high LDL-C baboons using *in silico* methods.

For differentially expressed miRNAs responsive to the HCHF diet, we observed a substantial (> 90%) overlap between targets identified in human and rhesus macaque genomes (Table 5). Because the genome assembly of baboon is in draft form, we used the rhesus genome to identify predicted gene targets. Baboon is closely related to rhesus macaque with 98% sequence identity [74]. This conservation of a large percentage of miRNA target sites between human and rhesus and the high sequence identity between rhesus and baboon suggests that our findings in baboons may translate to humans given that our data constitute sequences that are identical between human and baboon genomes. Nonetheless, a recent study [75] has shown that evolutionary changes in miRNA binding sites, may inhibit or enhance the effect of miRNAs on gene expression regulation. The study by Richardson et al. revealed that the *PLIN4* 3’ UTR has undergone nucleotide substitution allowing the binding of miR-522 for human individuals with A but not G allele. The miR-522 binding site with A allele was not found in Neanderthal and non-human primates suggesting a recent evolutionally change. Until an annotated baboon genome sequence is available we will not be able to assess conservation of miRNA targets sites between baboon and human and baboon and rhesus.

In this study, we observed many-genes-to-many-miRNAs regulation modules, suggesting that many genes are coordinately and cooperatively regulated by miRNAs. Genes targeted by multiple miRNAs are tightly regulated and may show graded expression in response to expression of different miRNAs. In addition, genes tightly regulated by redundant miRNAs may be an indication of their central role in the cell. We observed that, on average, about 2,000 genes are targeted by a single miRNA family, which presents daunting challenges for the development of a pharmaceutical agent with specific target. Moreover miRNA target prediction revealed that some genes are cooperatively targeted by miRNAs differing in their response to HCHF diet; i.e., one miRNA was up-regulated while the other was down-regulated in response to the HCHF diet, but both miRNAs have the same target. For example, *LDLRAP1* (low density lipoprotein receptor adapter protein 1, which interacts with LDL-C receptor) is targeted by miR-27a and -26a that are up- and down-regulated, respectively, in response to the HCHF diet. The molecular interactions of these miRNAs with *LDLRAP1* are not known, but it is plausible this phenomenon may be an inherent mechanism to balance gene expression levels. miR-27a and 26a are differentially regulated and implicated in adipogenesis [76,77].

We estimate that differentially expressed miRNAs in our study targeted 9% of the total genes implicated in atherosclerosis [78,79]. The following miRNA targets are included among those potentially associated with atherosclerosis: 1) *LDLR*, a cell surface protein involved in receptor-mediated endocytosis of LDL-C. Mutations in this gene cause the autosomal dominant disorder familial hypercholesterolemia [80] and increased carotid artery intima-media thickness [81]. 2) *VLDLR* (very low density lipoprotein receptor) plays important roles in VLDL triglyceride metabolism [82]. 3) *LIPA* (lipase A precursor) catalyzes the hydrolysis of cholesterol esters and triglycerides in lysosomes, and mutations in the gene is associated with impaired endothelial function in CVD [83]. Other targets of interests include *PTEN* (phosphatase and tensin homolog), which functions as a tumor suppressor by negatively regulating intracellular levels of phosphatidylinositol-3,4,5-trisphosphate, substrates for *AKT* (tyrosine kinase B protein) in the intracellular *AKT/PKB* signaling pathway, and *TENC1* (tensin-like C1 domain containing phosphatase), which is thought to act in a similar manner as *PTEN* in depriving substrates for *AKT*[84]. Further, we observed that *ACVR1B* (activin A receptor, type IB isoform c precursor) is considerably targeted by both novel and known miRNAs. *ACVR1B* is well-conserved in primates including human, rhesus, and orangutan, but relatively less-conserved in chimpanzee. *ACVR1B* is a receptor for activin growth factor, which belongs to *TGF-beta* (transforming growth factor-beta) superfamily. *TGF-beta* is involved and implicated in inflammatory processes, such as atherosclerosis in arteries [85]. Altogether this study identifies potential atherogenic candidate genes, targeted by differentially expressed baboon miRNAs. Identifying the roles of miRNAs and their target genes and signaling pathways in atherosclerosis will be critical for future research. miRNAs represent new regulatory factors for cardiovascular biology and are an emerging novel class of therapeutic targets for CVD.

Although identification of miRNA gene targets is a key step in elucidating miRNA functional mechanisms, comprehensive identification of targets still presents a daunting challenge. There are many bioinformatics tools available to predict miRNA targets; however, consistency among different platforms is limited [86]. In this study, we employed TargetScan, which is the most widely used tool in target prediction [87]. Because of the limitation of the high-throughput *in-silico* prediction, experimental validations of the miRNA-mRNA interaction are necessary. The experiments include reporter gene assay and loss- or gain-of-function of miRNA to measure target repression. However, these validation experiments are cumbersome, and, as a result, there are only a few validated miRNA targets. For example, our results generated using IPA indicate only 1% of the total miRNA targets have been validated. Recently, a high-throughput and sensitive technique for identification and quantification of miRNA targets has been reported [88]. The technique involves *in-situ* co-immunoprecipitation of mRNA associated with RISC, loaded with a specific miRNA, and subsequent deep sequencing. The technique, RISC-Seq, is a modification of a previous approach that utilized microarray profiling [89] and has been further optimized [90]. There is no doubt the technique provides important insight into determining the full spectrum of miRNA-mRNA interactions. However, the method is not yet fully optimized, and the modifications suggested by Matkovich et al. [90] present challenges due to over-expression of Argonaute2 protein in RISC complex, which may induce out-of-context cell state. Second, the technique does not address all outcomes of functions of miRNAs; a substantial number of miRNAs act by silencing the translation machinery and epigenetic modifications. Thus, until the RISC-Seq approach is fully optimized, *in-silico* prediction and basic experimental validations will remain useful approaches in deciphering the miRNA-mRNA interactions.

## Conclusion

We have sequenced baboon liver small RNA samples from half-sibs discordant for serum LDL-C and were fed chow and HCHF diets. We identified 517 baboon miRNAs including 490 that are identical to human and 27 novel miRNAs. The list of miRNAs reported in this study will contribute to the public database for small RNAs. Further, we identified 30 differentially expressed miRNAs in response to a HCHF challenge diet in two distinct baboon phenotypes previously characterized for extreme responses to dietary fat. Six of these differentially expressed miRNAs have been previously identified as regulators of genes that play roles in CVD. We also identified miRNA variants and other non-coding RNAs differentially expressed in response to the HCHF diet. Analysis of target genes of the differentially expressed miRNAs showed that 9% of the currently annotated CVD-related genes are predicted to be regulated by these miRNAs. Future studies that include gene sequencing in these samples to identify all expressed genes and gene variants in combination with gene/miRNA interactions in these LDL-C responders will take us closer to understanding the genetic and epigenetic networks that underlie LDL-C response to dietary fat and cholesterol, thus providing potential central genetic targets for miRNA-based therapeutic agents to regulate LDL-C.

## Methods

### Animals

Baboons (*Papio hamadryas*) were maintained by the Southwest National Primate Research Center (SNPRC) at the Texas Biomedical Research Institute (Texas Biomed), which is accredited by the Association for Assessment and Accreditation of Laboratory Animal Care International. Experimental protocols were approved by the Institutional Animal Care and Use Committee and were conducted under the supervision of Southwest National Primate Research Center staff veterinarians.

### Selection of baboon sib-pairs discordant for LDL-*C*

Based on phenotypic and genotypic analysis of 951 pedigreed baboons, we identified three half-sib-pairs with contrasting phenotypes for LDL-C as described [91]. The sib-pairs differed by at least two standard deviations for LDL-C serum concentrations. In addition, members of each selected sibling pair were discordant for at least 1 marker loci within the support interval for an LDL-C QTL on baboon chromosome 4 [92].

### High-cholesterol, high-fat (HCHF) dietary challenge

The dietary challenge protocol has been described elsewhere [93,94]. Briefly, liver biopsies were collected from the half-sib animals at baseline while consuming a basal diet (chow) low in fat (4% of calories) and cholesterol (0.03 mg/kcal). The animals were then fed the HCHF diet, high in fat (40% of calories) and cholesterol (1.7 mg/kcal), for 7 weeks, and liver biopsies were collected. Tissue samples were snap-frozen in liquid nitrogen and stored at −80°C. Table 8 shows the mean and standard deviation of the LDL-C levels in low and high LDL-C baboons on chow and HCHF diets.

**Table 8:** The mean (mg/dL) and standard deviation of the LDL-C levels in low and high LDL-C baboons on chow and HCHF diets

**Diet**	**Chow**	**HCHF**
High LDL-C Responders	43.7 (15.01)	114.3 (19.5)
Low LDL-C Responders	19.3 (8.1)	31.3 (2.5)

Standard deviations are indicated in parenthesis.

### Liver biopsy collection

Baboons were sedated with ketamine (10 mg/kg), given atropine (0.025 mg/kg) and intubated. Anesthesia was induced and maintained with isoflurane (1-2%). Blood pressure was measured by automated arm cuff (Collin) and oxygen saturation, heart rate, and respiration were monitored by pulse oximetry. Biopsies were collected from the left lobe of the liver using Temno Evolution Needle, gauge 14 (CareFusion, San Diego, CA). During post biopsy recovery analgesia was provided in the form of Stadol, 0.15 mg/kg, bid, for 3 days and ampicillin, 25 mg/day for 10 days.

### Small RNA library preparation

miRNA-enriched fractions were isolated from 10 mg of baboon liver (n = 12) using the mirVana^TM^ miRNA Isolation Kit (Ambion) according to the manufacturer’s protocol and stored at −80°C until further use. Small RNA libraries were prepared from the enriched fractions with Illumina Small RNA Prep Kit v1.5 following the manufacturer’s instructions. Briefly, the Illumina 3’ adapter (5’-ATCTCGTATGCCGTCTTCTGCTTGT) was ligated to 2 μg of enriched small RNAs using T4 RNA truncated ligase (New England BioLabs) and 15% PEG at 22°C for 2 hrs. Then, the Illumina 5’ adapter (5’-GTTCAGAGTTCTACAGTCCGACGATC) was ligated onto the 3’ adapter-linked RNA using T4 RNA ligase (New England BioLabs) for 1 hr at 20°C. The 5’ and 3’ adapter-linked RNA was converted to a single-stranded cDNA using SuperScript II Reverse Transcriptase (Invitrogen) and Illumina’s RT-Primer (5’-CAAGCAGAAGACGGCATACGA) following the manufacturer’s protocol. The cDNA was PCR-amplified with Hotstart Phusion DNA Polymerase using the following profile: denaturation at 98°C for 30s, 15 cycles of 98°C for 10s, 60°C for 30s, 72°C for 15 s, and a final extension at 72°C for 10 min.

The PCR product was purified on a 6% TBE urea polyacrylamide gel (Invitrogen). The gel was stained with ethidium bromide and visualized under UV light. The gel band corresponding to 93–100 bp was excised, passed through a bottom-perforated 0.5 ml tube and eluted in 1X Elution Buffer following the manufacturer’s protocol. Notably, while the average length of a miRNA is 22 nucleotides (nts), the excised band corresponds to the total length of the adapter-ligated miRNAs. The resulting gel slurry was spun in a Spin-X filter and RNA precipitated with 2ul of glycogen, 10ul of 3 M-ammonium acetate and 325 μl of 100% cold ethanol. The mixture was centrifuged, and the resulting pellet washed with 70% ethanol. The dried RNA pellet was suspended in 10 μl of Resuspension Buffer (Illumina).

### Next generation sequencing of small RNAs

Small RNA sequencing was performed using Illumina’s Genome Analyzer (GAIIx). Diluted cDNA (10 nmol/L) was amplified, sequenced, and single-read clusters generated using cBot Cluster Generation Kit and TruSeq SBS Kit v5 (36 cycles). For the control sample, we used PhiX Control Kit v3 (Illumina).

### Analysis of sequence reads

#### Reads filtering and adapter trimming

miRNA sequence reads were analyzed using mirTools [95], an interactive web-based tool for analyzing deep sequencing reads. Briefly, the small RNA sequence reads were filtered to exclude low-quality reads and 3’/5’ adapter- and polyA-sequences. The reads were trimmed off the 3’ adapter sequences using a custom *perl* script. The resulting clean full-length reads were formatted into a non-redundant FASTA file. Each sequence tag in the file contains the number of reads represented by the tag.

#### Small RNA annotation

The resulting sequence tags (18–30 nts) were mapped onto the human genome sequence (hg18) using the SOAP2 program [96] embedded in mirTools. Subsequently, the reads were aligned against various databases including miRBase [97], Rfam [98], RepeatMasker [99], and the reference genome transcriptome database. Consequently the sequence tags were classified as a known miRNA, a degraded fragment from non-coding RNA, or RNA derived from repeat or coding sequences. Sequences that did not conform to any of the categories but mapped to a unique location on the human genome were termed “unclassified.”

#### Discovery of novel miRNAs

The prediction of novel miRNAs was performed by miRDeep software [54] and associated RNAfold software [100], incorporated in mirTools using default settings. Unclassified tags were used to predict candidate novel miRNAs. Using RNAfold software, a sequence fragment containing 100 nts flanking each side of the locus aligning to an unclassified tag was excised from the genome sequence. RNAfold software folds and performs computational assessment of the fragment to ensure that there is a minimum of 14 base pairings between the mature and star sequences of the hairpin. Because false positive hairpins can be formed from different types of small RNAs in the genome, miRDeep software performs further stringent analysis to test whether reads aligning to a potential miRNA precursor (pre-miRNA) are consistent with the Dicer processing signature to simulate miRNA biogenesis in the cell; presence of 2-nt 3’ overhangs and positional alignment of the reads on mature, star, or loop of the precursor sequence. Extracted sequences that do not have a Dicer signature and fold into a hairpin were discarded. An unclassified sequence tag is considered novel miRNA if it aligns to a candidate pre-miRNA. Sequences with dominantly abundant reads were denoted as mature miRNA and its complementary sequence as miRNA*.

### Detection of differential expression of small RNAs

miRNAs and other non-coding RNAs differentially expressed between chow and HCHF samples were determined by assessing the read counts as described [95]. A sequence tag consists of one or more reads, which reflects the number of times the tag was sequenced, hence the expression level. We normalized an expression level of a sequence tag, within and between samples, by dividing the absolute read count by the total read count of the sample and multiplied by 1,000,000. The normalized count corresponds to the relative expression level of each tag. Normalized expression data generated from animals on chow and challenge diets were used to determine differential expression levels in each phenotype. A two-tailed student *t*-test was performed to test for significant expression levels for miRNAs expressed on both diets and Welch’s *t*-test, assuming unequal variance, was performed for miRNAs with expression detected in only one of the two groups. A small non-coding RNA was considered differentially expressed between samples when the p-value was ≤ 0.05, and multiple testing adjustments applied, where appropriate, using False Discovery Rate [101].

### Target prediction

We identified potential targets of diet responsive differentially expressed miRNAs in low LDL-C and high LDL-C responders using TargetScan/Base Release 5.1 ( http://www.targetscan.org/) and Ingenuity Pathway Analysis ( http://www.ingenuity.com) tools. We identified novel miRNA targets using TargetScan [102], employing the 7-mer seed-region of each miRNA sequence to customize prediction of targets.

## Abbreviations

CVD: Cardiovascular disease; HDL-C: High-density lipoprotein cholesterol; LDL-C: Low-density lipoprotein cholesterol; ox-LDL-C: Oxidized LDL-C; BCL2: B-cell lymphoma protein 2 beta isoform; microRNAs: miRNAs; PEG: Polyethylene glycol; HCHF: High-cholesterol, high-fat; SOAP: Short oligonucleotide alignment program; nts: Nucleotides; siRNAs: Small interfering RNAs; sno RNAs: Small nucleolar RNAs; snRNAs: Small nuclear RNAs; tRNAs: Transfer RNAs; RSC: RNA-induced silencing complex; miRNA*: miRNA star; *TENC1*: Tensin like C1 domain containing phosphatase; *VLDLR*: Very low density lipoprotein receptor; *LDLR*: Low density lipoprotein receptor; *LIPA*: Lipase A precursor; *PTEN*: Phosphatase and tensin homolog; *AKT*: Tyrosine kinase B protein; *TENC1*: Tensin-like C1 domain containing phosphatase; *ACVR1B*: Activin A receptor, type IB isoform c precursor; *TGF-beta*: Transforming growth factor-beta; cDNA: Complementary DNA; FDR: False Discovery Rate.

## Competing interests

The authors declare that they have no competing interests.

## Authors’ contributions

GMK, JPG and LAC participated in the conception and design of the experiments and performed data analyses. All authors contributed to writing this manuscript and all have read and approved the final manuscript.

## Supplementary Material

Additional file 1 A Microsoft Excel file containing human ortholog miRNAs expressed in livers of low and high LDL-C baboons on chow and HCHF diets. Click here for file

Additional file 2 A Microsoft Excel file containing baboon novel mRNAs expressed in livers of low and high LDL-C baboons on chow and HCHF diets. Click here for file

Additional file 3 A Microsoft Word file with supplemental tables. Click here for file

Additional file 4 A Microsoft Excel file containing expressed other non-coding RNAs in response to HCHF diet in high LDL-C baboon livers. Click here for file
