# Supplementary material for: Differential microRNA response to a high-cholesterol, high-fat diet in livers of low and high LDL-C baboons
Source: BMC Genomics. 2012 Jul 18;13:320. doi: 10.1186/1471-2164-13-320 (PMC3536563; doi:10.1186/1471-2164-13-320)
Supplement: Additional file 3 — A Microsoft Word file with supplemental tables. [file 1471-2164-13-320-S3.doc]

**Table S1.** Differentially expressed miRNAs in response to the HCHF diet

| **miRNA ID** | **Hair- pin arm** | | **Sequence** | **p-value** | | | **Fold change** | **Change** |
| --- | --- | --- | --- | --- | --- | --- | --- | --- |
| High LDL-C responders | | | | | | | |  |
| hsa-miR-125b | 5p | | UCCCUGAGACCCUAACUUGUGA | | 0.01 | -17.3 | | Down |
| hsa-miR-16-2* | 3p | | CCAAUAUUACUGUGCUGCUUUA | | 0.00 | -9.4 | | Down |
| hsa-miR-190 | 5p | | UGAUAUGUUUGAUAUAUUAGGU | | 0.03 | 140.4 | | Up |
| hsa-miR-191* | 3p | | GCUGCGCUUGGAUUUCGUCCCC | | 0.03 | -2.0 | | Down |
| hsa-miR-194* | 3p | | CCAGUGGGGCUGCUGUUAUCUG | | 0.02 | -31.2 | | Down |
| hsa-miR-19b | 3p | | UGUGCAAAUCCAUGCAAAACUGA | | 0.02 | -7.8 | | Down |
| hsa-miR-200b | 3p | | UAAUACUGCCUGGUAAUGAUGA | | 0.01 | -47.7 | | Down |
| hsa-miR-221 | 3p | | AGCUACAUUGUCUGCUGGGUUUC | | 0.02 | -67.4 | | Down |
| hsa-miR-222 | 3p | | AGCUACAUCUGGCUACUGGGU | | 0.01 | -26.0 | | Down |
| hsa-miR-326 | 3p | | CCUCUGGGCCCUUCCUCCAG | | 0.03 | -5.4 | | Down |
| hsa-miR-369-3p | 3p | | AAUAAUACAUGGUUGAUCUUU | | 0.05 | 229.8 | | Up |
| hsa-miR-425* | 3p | | AUCGGGAAUGUCGUGUCCGCCC | | 0.04 | -91.4 | | Down |
| hsa-miR-487a | 3p | | AAUCAUACAGGGACAUCCAGUU | | 0.02 | 2.2 | | Up |
| hsa-miR-513b | 5p | | UUCACAAGGAGGUGUCAUUUAU | | 0.04 | -4.3 | | Down |
| hsa-miR-551b | 3p | | GCGACCCAUACUUGGUUUCAG | | 0.04 | -13.0 | | Down |
| hsa-miR-654-5p | 5p | | UGGUGGGCCGCAGAACAUGUGC | | 0.00 | -13.1 | | Down |
| hsa-miR-660 | 5p | | UACCCAUUGCAUAUCGGAGUUG | | 0.03 | -126.7 | | Down |
| hsa-miR-874 | 3p | | CUGCCCUGGCCCGAGGGACCGA | | 0.04 | -8.9 | | Down |
| hsa-miR-93 | 5p | | CAAAGUGCUGUUCGUGCAGGUAG | | 0.00 | -410.7 | | Down |
|  |  | |  | |  |  | |  |
| Low LDL-C responders | | | | | | | | |
| hsa-miR-26a-1* | | 3p | CCUAUUCUUGGUUACUUGCACG | | 0.01 | | -1.9 | Down |
| hsa-miR-27a* | | 5p | AGGGCUUAGCUGCUUGUGAGCA | | 0.02 | | 2.5 | Up |
| hsa-miR-301b | | 3p | CAGUGCAAUGAUAUUGUCAAAGC | | 0.01 | | -1.2 | Down |
| hsa-miR-3154 | | 3p | CAGAAGGGGAGUUGGGAGCAGA | | 0.00 | | 1.1 | Up |
| hsa-miR-338-5p | | 5p | AACAAUAUCCUGGUGCUGAGUG | | 0.05 | | -4.7 | Down |
| hsa-miR-34b | | 3p | CAAUCACUAACUCCACUGCCAU | | 0.00 | | -1.1 | Down |
| hsa-miR-377* | | 5p | AGAGGUUGCCCUUGGUGAAUUC | | 0.04 | | -1.6 | Down |
| hsa-miR-380* | | 5p | UGGUUGACCAUAGAACAUGCGC | | 0.03 | | -1.1 | Down |
| hsa-miR-486-5p | | 5p | UCCUGUACUGAGCUGCCCCGAG | | 0.02 | | -1.2 | Down |
| hsa-miR-887 | | 3p | GUGAACGGGCGCCAUCCCGAGG | | 0.03 | | -18.8 | Down |
|  | |  |  | |  | |  |  |
| Shared between the phenotypes | | | | | | | | |
| hsa-miR-29a | | 3p | UAGCACCAUCUGAAAUCGGUUA | 0.01 | | | -5260.1 | Down |
| hsa-miR-29b | | 3p | UAGCACCAUUUGAAAUCAGUGUU | 0.02 | | | -14.4 | Down |
| hsa-miR-30d* | | 3p | CUUUCAGUCAGAUGUUUGCUGC | 0.05 | | | -21.4 | Down |

**Table S2. miRNAs expressed on only one diet**

| **Mean read count in low LDL-C animals** | | | |
| --- | --- | --- | --- |
|  | **Chow** | **HCHF** | **p**  **value** |
| hsa-miR-301b | 1.22 | 0.00 | 0.007 |
| hsa-miR-767-5p | 0.39 | 0.00 | 0.184 |
| hsa-miR-3609 | 0.39 | 0.00 | 0.184 |
| hsa-miR-1226 | 0.00 | 0.92 | 0.184 |
| hsa-miR-299-3p | 0.00 | 0.92 | 0.184 |
| hsa-miR-641 | 0.48 | 0.00 | 0.196 |
| hsa-miR-3200-3p | 1.55 | 0.00 | 0.239 |
| hsa-miR-574-3p | 0.45 | 0.00 | 0.295 |
| hsa-miR-454* | 0.49 | 0.00 | 0.305 |
| hsa-miR-3202 | 0.00 | 0.15 | 0.423 |
| hsa-miR-1293 | 0.00 | 0.15 | 0.423 |
| hsa-miR-320c | 0.00 | 0.22 | 0.423 |
| hsa-miR-181a* | 0.00 | 0.22 | 0.423 |
| hsa-miR-224 | 1.89 | 0.00 | 0.423 |
| hsa-miR-106a | 0.00 | 0.22 | 0.423 |
| hsa-miR-758 | 0.00 | 0.22 | 0.423 |
| hsa-miR-378b | 0.00 | 0.45 | 0.423 |
| hsa-miR-1246 | 0.00 | 0.22 | 0.423 |
| hsa-miR-600 | 0.00 | 0.22 | 0.423 |
| hsa-miR-933 | 0.00 | 0.22 | 0.423 |
| hsa-miR-615-3p | 0.47 | 0.00 | 0.423 |
| hsa-miR-1243 | 0.09 | 0.00 | 0.423 |
| hsa-miR-105* | 0.19 | 0.00 | 0.423 |
| hsa-miR-200c* | 0.36 | 0.00 | 0.423 |
| hsa-miR-3190 | 0.19 | 0.00 | 0.423 |
| hsa-miR-302a | 0.09 | 0.00 | 0.423 |
| hsa-miR-452* | 0.09 | 0.00 | 0.423 |
| hsa-miR-218-1* | 0.28 | 0.00 | 0.423 |
| hsa-miR-138-1* | 0.19 | 0.00 | 0.423 |
| hsa-miR-23a* | 0.28 | 0.00 | 0.423 |
| hsa-miR-526b* | 0.28 | 0.00 | 0.423 |
| hsa-miR-1288 | 0.09 | 0.00 | 0.423 |
| hsa-miR-205 | 0.09 | 0.00 | 0.423 |
| hsa-miR-199a-5p | 0.36 | 0.00 | 0.423 |
| hsa-miR-3120 | 0.36 | 0.00 | 0.423 |
| hsa-miR-3605-5p | 0.09 | 0.00 | 0.423 |
| hsa-miR-196a* | 1.14 | 0.00 | 0.423 |
| hsa-miR-367 | 0.09 | 0.00 | 0.423 |
| hsa-miR-3129 | 0.20 | 0.00 | 0.423 |
| hsa-miR-525-5p | 0.00 | 0.30 | 0.423 |
| hsa-miR-219-5p | 0.28 | 0.00 | 0.423 |
|  |  |  |  |
|  |  |  |  |
|  | **Chow** | **HCHF** | **p-value** |
| hsa-miR-3934 | 0.09 | 0.00 | 0.423 |
| hsa-miR-302d | 0.09 | 0.00 | 0.423 |
| hsa-miR-450a | 0.09 | 0.00 | 0.423 |
| hsa-miR-517c | 0.19 | 0.00 | 0.423 |
| hsa-miR-2355-5p | 0.09 | 0.00 | 0.423 |
| hsa-miR-590-3p | 0.28 | 0.00 | 0.423 |
| hsa-miR-520d-5p | 0.19 | 0.00 | 0.423 |
| hsa-miR-767-3p | 0.09 | 0.00 | 0.423 |

| **Mean count in High LDL-CLDL-C animals** | | | |
| --- | --- | --- | --- |
|  | **Chow** | **HCHF** | **p value** |
| hsa-miR-487a | 0.00 | 2.19 | 0.019 |
| hsa-miR-378 | 0.00 | 1.74 | 0.185 |
| hsa-miR-218-1* | 0.00 | 0.26 | 0.188 |
| hsa-miR-517c | 1.17 | 0.00 | 0.196 |
| hsa-miR-2114 | 0.00 | 0.93 | 0.204 |
| hsa-miR-372 | 1.84 | 0.00 | 0.208 |
| hsa-miR-760 | 0.00 | 0.19 | 0.208 |
| hsa-miR-320b | 0.00 | 0.19 | 0.208 |
| hsa-miR-432* | 0.00 | 0.43 | 0.224 |
| hsa-miR-676* | 0.00 | 0.68 | 0.235 |
| hsa-miR-3191 | 0.00 | 0.40 | 0.246 |
| hsa-miR-200a* | 0.00 | 0.54 | 0.291 |
| hsa-miR-3155 | 0.00 | 0.35 | 0.301 |
| hsa-miR-3074 | 0.00 | 0.35 | 0.301 |
| hsa-miR-574-3p | 0.00 | 0.35 | 0.301 |
| hsa-miR-371-5p | 0.00 | 0.35 | 0.301 |
| hsa-miR-182* | 0.00 | 0.57 | 0.423 |
| hsa-miR-198 | 0.00 | 0.07 | 0.423 |
| hsa-miR-320c | 0.00 | 0.07 | 0.423 |
| hsa-miR-3909 | 0.00 | 0.07 | 0.423 |
| hsa-miR-199a-5p | 0.00 | 0.07 | 0.423 |
| hsa-miR-3165 | 0.00 | 0.07 | 0.423 |
| hsa-miR-3200-3p | 0.00 | 0.07 | 0.423 |
| hsa-miR-641 | 0.00 | 0.07 | 0.423 |
| hsa-miR-3940 | 0.00 | 0.07 | 0.423 |
| hsa-miR-2355-5p | 0.00 | 0.07 | 0.423 |
| hsa-miR-224 | 0.00 | 0.57 | 0.423 |
| hsa-miR-412 | 0.00 | 0.14 | 0.423 |
| hsa-miR-187* | 0.00 | 0.14 | 0.423 |
| hsa-miR-181a | 0.00 | 0.14 | 0.423 |
| hsa-miR-378b | 1.72 | 0.00 | 0.423 |
| hsa-miR-670 | 0.57 | 0.00 | 0.423 |
| hsa-miR-1298 | 0.00 | 0.35 | 0.423 |
| hsa-let-7c* | 0.00 | 0.21 | 0.423 |
| hsa-miR-1243 | 0.00 | 0.28 | 0.423 |
| hsa-miR-4306 | 0.00 | 0.12 | 0.423 |
| hsa-miR-1306 | 0.00 | 0.23 | 0.423 |
| hsa-miR-653 | 0.00 | 0.12 | 0.423 |
| hsa-miR-452* | 0.00 | 0.28 | 0.423 |
| hsa-miR-149* | 0.48 | 0.00 | 0.423 |
| hsa-miR-302a* | 0.00 | 0.28 | 0.423 |
| hsa-miR-181a* | 0.00 | 0.28 | 0.423 |
| hsa-miR-138-2* | 0.48 | 0.00 | 0.423 |
| hsa-miR-196a* | 0.00 | 0.28 | 0.423 |
| hsa-miR-1251 | 0.69 | 0.00 | 0.423 |
| hsa-miR-218-2* | 0.48 | 0.00 | 0.423 |
| hsa-miR-202 | 0.00 | 0.28 | 0.423 |
| hsa-miR-933 | 0.00 | 0.12 | 0.423 |
| hsa-miR-3610 | 0.00 | 0.28 | 0.423 |
| hsa-miR-1197 | 0.00 | 0.21 | 0.423 |
| hsa-miR-1234 | 0.69 | 0.00 | 0.423 |
| hsa-miR-1293 | 0.00 | 0.12 | 0.423 |
